# Measuring mental well-being in Sri Lanka: validation of the Warwick Edinburgh Mental Well-being Scale (WEMWBS) in a Sinhala speaking community

**DOI:** 10.1186/s12888-022-04211-8

**Published:** 2022-08-24

**Authors:** B. P. R. Perera, A. Caldera, P. Godamunne, S. Stewart-Brown, A. R. Wickremasinghe, R. Jayasuriya

**Affiliations:** 1 Department of Public Health, Faculty of Medicine, University of Kelaniya, Thalagolla Road 11010, P.O. Box 6, Ragama, Sri Lanka; 2 Warwick Medical School, University of Warwick, Coventry, UK; 3 School of Population Health, University of New South Wales, Randwick, NSW 2052 Australia

**Keywords:** WEMWBS Sinhala version, Validation, Mental well-being, Sri Lanka

## Abstract

**Background:**

Well-being is an important aspect of people’s lives and can be considered as an index of social progress. The Warwick Edinburgh Mental Well-being scale (WEMWBS) was developed to capture subjective mental well-being. It is a widely tested measure of mental well-being at the population level and has 14 items and a short-form with 7 items. This study was carried out to culturally validate and adapt the WEMWBS among a Sinhala speaking population in Sri Lanka.

**Methods:**

A forward and backward translation of the scale into Sinhala was done followed by a cognitive interview. The translated and culturally adapted scale and other mental health scales were administered to a sample of 294 persons between the ages of 17–73 using a paper-based version (*n* = 210) and an online survey (*n* = 84). Internal consistency reliability and test–retest reliability were tested. Construct validity, and convergent and discriminant validity were assessed using the total sample.

**Results:**

The translated questionnaire had good face and content validity. Internal consistency reliability was 0.91 and 0.84 for the 14-item and 7-item scales, respectively. Test–retest reliability over two weeks was satisfactory (Spearman *r* = 0.72 *p* < 0.001). Confirmatory factor analysis supported a one factor model. Convergent validity was assessed using WHO-5 well-being index (Spearman *r* = 0.67, *p* < 0.001), Patient Health Questionnaire (PHQ-9) (Spearman *r* = (-0.45), *p* < 0.001) and Kessler psychological distress scale (K10) (Spearman *r* = (-0.55), *p* < 0.001).

**Conclusions:**

The translated and culturally adapted Sinhala version of the WEMWBS has acceptable psychometric properties to assess mental well-being at the population level among the Sinhala speaking population in Sri Lanka.

**Supplementary Information:**

The online version contains supplementary material available at 10.1186/s12888-022-04211-8.

## Introduction

The concept of mental well-being is a relative newcomer to public health and public mental health more specifically. It is important because it is predictive of better overall health, fewer disorders and physical disabilities, and less use of health and social care [1, 2]. Well-being is also an important aspect of people’s lives in its own right and can be considered as an index of social progress [3, 4].

Mental well-being has been conceptualised by the WHO as a state in which individuals realize their abilities, can cope with the normal stresses of life, can work productively and fruitfully, and are able to contribute to their community [5]. This is a primarily eudaimonic definition focusing on functioning. Others have conceptualised well-being in hedonic terms using measures of happiness or life satisfaction [6]. Yet, others see mental well-being as comprising both eudaimonic and hedonic perspectives and describe it as feeling good and functioning well [7].

With the development of mental health policies in most countries in Asia, there is a need for validated indicators to monitor mental well-being. The mental health policy of Sri Lanka was gazetted in 2005 [8]. Its vision is to provide a comprehensive, community-based, affordable and accessible service to promote and optimise mental well-being among the citizens of the country. However, the lack of a validated tool with good psychometric properties was a major obstacle to assess mental health status in the country and to monitor the effect of interventions and services provided.

Following a review of measures available for this purpose we chose to translate and validate the long- and short-versions of the Warwick-Edinburgh Mental Wellbeing Scales (S/WEMWBS) in the majority Sinhala speaking population of Sri Lanka.

The Warwick Edinburgh Mental Well-being Scale (WEMWBS) addresses the third definition of mental well-being described above covering both hedonic and eudaimonic aspects of mental well-being. It is a 14-item scale developed in the UK to meet the need for a psychometrically robust measure of positive mental health for use in population surveys, epidemiological studies and evaluation of interventions aiming to improve mental well-being [9]. The hedonic aspect includes the subjective experience of cheerfulness, optimism, and feeling relaxed and the eudaimonic aspect includes items such as feeling useful, thinking clearly and good interpersonal relationships [9]. A key feature of WEMWBS is that all items are phrased positively and all relate to a positive aspect of mental health which increases acceptability among particpants.

The original validation of WEMWBS in the UK showed a single underlying factor and high reliability and validity in general population samples [9]. The 7-item short version of WEMWBS was subsequently resolved [10]. Both the short- and original- versions have been tested in many age groups and settings:- student [10, 11] and adult samples [9, 12, 13], clinical and non-clinical settings [14–16], veterinary professionals [12], nursing trainees [17], general managers in a hotel chain [18] and healthcare professionals [19]. An early large focus group qualitative study in the UK demonstrated validity of the scale in culturally varied populations – Pakistani and Chinese. The scales have consistently shown good psychometric properties, including responsiveness, an essential property in scales which could be used for intervention evaluation [20]. They have also been benchmarked against commonly used scales of mental illness [21, 22].

WEWMBS proved very popular in the UK where it was developed because it is well liked by academics, practitioners and study participants and is easy to apply. Population distributions approximate to normal facilitating epidemiological analyses and monitoring, and the scales' sensitivity to change make them measures of choice for evaluation of large- and small-scale interventions. The scales are being used to monitor mental well-being at national level in all three UK countries and are required for contract monitoring in some local and national government contracts.

WEMWBS is now used in 50 countries world-wide. It has been adapted into languages and tested among different cultures and populations, in western countries [9, 11, 18, 21, 23–27] and non-western cultures [28, 29]. Some studies in Asia [17, 28–30] as well as in non-Asian countries [31] have found a second minor factor in addition to the strong main factor model.

The mental health policy of Sri Lanka has a vision to promote and optimise mental well-being among the citizens of the country [8]. This study aimed to validate the long- and short-versions of the WEMWBS scale in the majority Sinhala speaking group in Sri Lanka so that it may be used to assess mental well-being at the population level.

## Methods

### Participants and data collection

This was a general population sample which includes people with mental illness. All consenting participants between 17–73 years who can read Sinhala were enrolled in the study regardless of their mental health status. No incentives were provided to the participants. The final sample included 294 participants comprising a community sample (*n* = 210) in a sub-division of the Gampaha district (Ragama Medical Officer of Health area (MOH)) demarcated for population health services using a paper-based questionnaire. Gampaha District is divided into16 MOH areas. Ragama MOH area is a semi-urban area with a population of approximately 79,596 in 2020 [32] Ragama MOH area has 14 Grama Niladhari (GN) divisions. One house was randomly selected from each Grama Niladhari area from a list of households in the GN division. After the selection of the first house, the next house on the immediate right of the first house was selected. When there were no more houses in a particular area a new starting point was selected and the process was repeated. The participants were visited at their homes and were invited to participate in the study. Only one participant was selected from a house. Where there were more than one eligible participant, the one with the highest educational level was chosen. The questionnaire was handed to participants following informed consent. Participants completed the questionnaire including 47 questions/items comprising the Sinhala versions of the 14-item WEMWBS, WHO-5, PHQ-9, K10 and questions on demographic characteristics. They returned it to the investigators on the same day or a subsequent day at visits to the house. At least two attempts were made to retrieve the completed questionnaires. Two of the investigators were present during data collection in the field. They were available to provide clarifications on the questionnaire if requested, but this happened rarely.

An online sample was selected using snowball sampling (*n* = 84). A Google form was shared among personal contacts who were Sinhala speaking Sri Lankans currently residing in Sri Lanka and were between 17–73 years. They were requested to share it with their contacts who fitted the eligible criteria and were currently residing in Sri Lanka. The Google form was shared via email, web and mobile platforms.

### Measures

#### Warwick Edinburgh Mental Well-being Scale (WEMWBS)

The WEMWBS consists of 14 positively phrased items rated on a 5-point Likert scale ranging from “none of the time” to “all of the time”. The well-being score is calculated by summing up the score of the 14 items. The global score can range from 14 to 70.

#### Translation of the WEMWBS into Sinhala

Two independent bilingual translators translated the 14-item scale from English to Sinhala. A combined version was back-translated into English by two independent translators who were fluent in both languages. The back-translated version was compared with the original English version. A pre-final translated version was provided to a committee of experts, consisting of two public health experts, two psychologists, a methodologist and translators for content validation. This process went through several iterations until the final back translation was similar to the original version. Selected participants from the general population comprising both sexes, different age categories and occupations were included in cognitive testing. Participants were asked to complete the scale, raise any issues of comprehension and given the chance to suggest alternative wording to the items to make them more comprehensive and meaningful. Issues faced during this process were discussed with the developer of the scale and resolved. The alternative translations suggested by the participants were included in the final version of the scale, where appropriate. The issues were related to item 4 (“I’ve been interested in other people”), item 9 (“I’ve been feeling close to other people”) and item 11 (“I’ve been able to make up my mind about things”). The final translations were approved by the copyright holders of the scale and permission was obtained to validate the tool in Sinhala.

### Other scales

#### WHO-5 well-being index

WHO-5 consists of five positively phrased statements concerning perceived well-being during the past two weeks. The raw score of an individual ranges from 0 to 25, multiplied by four to obtain a percentage. The questionnaire has been validated for a Sinhala speaking Sri Lankan population and has demonstrated acceptable psychometric properties [33].

#### Patient Health Questionnaire (PHQ-9)

The PHQ-9 depression module is a nine-item scale derived from the full 26-item PRIME-MD scale. The severity of the symptoms is rated on a scale from 0–3 corresponding to “not at all” to “nearly every day”, with a range from 0–27; higher values indicate increasing severity [34]. The PHQ-9 has been validated among a Sinhala speaking Sri Lankan population and has been found to have acceptable psychometric properties with a cut-off of 10 to screen for moderate depression [35].

#### Kessler psychological distress scale (K10)

K10 is a screening tool with 10 questions used in population surveys. The questions are scored on a 5-point Likert scale ranging from 0–4 and assess level of anxiety and depressive symptoms a person may have experienced in the past 4 weeks. A cut-off value of 16 has been suggested for referral for depression based on a validated questionnaire in Sinhala [36].

### Psychometric tests

#### Reliability

Cronbach’s alpha was used to assess internal consistency reliability; an α > 0.7 was considered to indicate satisfactory reliability [37]. Item-total correlation > 0.3 is another indicator of satisfactory reliability suggesting that the item is related to the overall scale [38]. Congeneric reliability was tested with confirmatory factor analysis (CFA) for standard factor loadings of 0.6 or above [39].

The test–retest reliability of the scale within 1–2 weeks was assessed in a sample of 60 participants using the Spearman rank correlation coefficient and the Intraclass Correlation Coefficient (ICC) based on the absolute agreement in a two-way mixed-method model. Reliability was classified as low (ICC < 0.5), moderate (ICC 0.5–0.75), high (ICC = 0.75–0.9) or excellent (ICC > 0.90) [40].

Ceiling and floor effects were assessed if 15% or more participants in the sample were achieving the highest or lowest possible score [41, 42].

#### Convergent and discriminant validity

We hypothesized that the WEMWBS will be positively correlated with WHO-5, and negatively correlated with K10 and PHQ-9. A correlation of >|0.5|was considered to indicate satisfactory convergent validity [43].

Known-group validity was assessed by comparing scores by age, gender, ethnicity, religion, educational level, and employment. We expected to find a higher mean well-being score among older persons, persons with higher education and those who were employed [44]. These hypotheses were tested using the Mann–Whitney-U test or the Kruskal Wallis test as appropriate.

#### Construct validity

The factorial validity of a single-factor model was tested by confirmatory factor analysis (CFA) with robust diagonally weighted least squares (DWLS) estimation using polychoric correlation matrix to account for ordinal items that are skewed [45, 46]. Model fit was considered acceptable using four approximate fit indices if: Chi-square test had a *p* < 0.05; Root Mean Square of Approximation (RMSEA) was < 0.06 with values up to 0.08 being considered as acceptable; Comparative Fit Index (CFI) and Tucker–Lewis Index (TLI) values > 0.90 and > 0.95, respectively; and Standard Root Mean Square Residual (SRMR) of < 0.05 [47]. Analyses were performed using Lavaan 0.6-5 and semPlot in R software [48–50].

#### Ethics

The Ethics Review Committee of the Faculty of Medicine, University of Kelaniya, Sri Lanka (Ref.No.P/128/06/2019) approved the study. Informed consent was obtained from the participants prior to the study. None of the participants were individually identified in the analyses.

## Results

### Sample characteristics

Two hundred and ten out of 240 paper-based questionnaires were returned (response rate = 87.5%). 84 persons completed the online questionnaire giving a total sample of 294. The ages of the respondents ranged from 17 to 73 years (mean = 33.7; standard deviation 13.2 years). The majority were female (68.7%), Sinhalese (96.3%) and Buddhist (70.7%). 59.9% of participants were educated above grade 13 and 47.5% were employed (Table 1).

**Table 1:** Socio-demographic profile of participants (*N* = 294)

Characteristic	N (%)
**Gender**^a^	
Male	88 (29.9)
Female	202 (68.7)
**Age(years)**	
15–24	79 (26.9)
25–44	159 (54.1)
45–74	56 (19.0)
**Religion**	
Buddhist	208 (70.8)
Hindu	03 (1.0)
Islam	05 (1.7)
Christian	78 (26.5)
**Ethnicity**	
Sinhala	283 (96.3)
Tamil	06 (2.0)
Moor	05 (1.7)
**Educational Level**	
School education only	118 (40.1)
Higher education	176 (59.9)
**Employment Status**	
Employed	139 (47.3)
Student	92 (31.3)
Not-employed	63 (21.4)

^a^4 persons had not indicated their gender

### Mental health status of the participants

Based on PHQ-9 scores, 21.7% were likely to have depressive symptoms; based on K10 scores, 23.1% had some form of psychological distress. There was no difference in WHO-5, PHQ-9 and K10 scores based on gender. Students had the lowest well-being scores assessed by WHO-5 and the highest depression score and psychological distress score, assessed by PHQ-9 and K10, respectively. PHQ-9 and K10 scores were similar among students and those not working. Those with a higher educational level had lower well-being scores. Among those who received higher education, 45.5% were students and 2.3% were currently not working (See Additional File 1).

### Psychometric properties

#### WEMWBS scale scores

The mean (± sd) scores for the 14-item and the 7-item scales were 52.5 (± 9.3) (median 53.5, IQR 48 to 60) and 25.9 (+ 4.8) (median 27, IQR 23 to 29), respectively. As expected, the correlation between the 14-item scale and the 7-item scale was high (Spearman *r* = 0.96; *p* < 0.001). All response categories were used by at least one person for each item (Table 2).

**Table 2:** Descriptive statistics of responses

**Item**	**Item wording**	**n**	**Percentages**					**Mean**	**Standard Deviation**	**Skewness**	**Kurtosis**
			None of the time	Rarely	Some of the time	Often	All of the time				
1	I’ve been feeling optimistic about the future	294	4.8	7.5	32.3	38.8	16.7	3.55	1.00	-0.57	0.15
2	I’ve been feeling useful	294	1.7	5.1	26.2	42.9	24.1	3.82	0.91	-0.59	0.26
3	I’ve been feeling relaxed	294	6.5	8.8	32.3	31.3	21.1	3.51	1.11	-0.48	-0.28
4	I’ve been feeling interested in other people	294	2.4	8.2	28.6	35.4	25.5	3.73	1.00	-0.49	-0.23
5	I’ve had energy to spare	294	4.4	9.5	31.3	34.4	20.4	3.56	1.05	-0.47	-0.21
6	I’ve been dealing with problems well	294	2.4	5.4	20.4	43.9	27.9	3.89	0.95	-0.83	0.60
7	I’ve been thinking clearly	294	1.7	7.5	27.9	40.1	22.8	3.74	0.94	-0.49	-0.09
8	I’ve been feeling good about myself	294	3.4	3.7	18.7	37.1	37.1	4.00	1.00	-1.03	0.87
9	I’ve been feeling close to other people	294	2.4	11.6	28.9	38.4	18.7	3.59	0.99	-0.41	-0.32
10	I’ve been feeling confident	294	2.0	4.4	15.6	37.1	40.8	4.10	0.95	-1.07	0.91
11	I’ve been able to make up mind about things	294	2.0	4.1	23.1	50.7	20.1	3.82	0.86	-0.79	1.04
12	I’ve been feeling loved	294	2.4	8.8	24.8	37.1	26.9	3.77	1.01	-0.58	-0.19
13	I’ve been interested in new things	294	2.7	9.9	22.1	36.4	28.9	3.78	1.05	-0.64	-0.22
14	I’ve been feeling cheerful	294	3.4	6.5	31.3	37.8	21.1	3.66	0.99	-0.54	0.11

*n- valid cases*

Between 16.7% and 40.8% of participants responded “all of the time” indicating a possible ceiling effect for the following items; the maximum score was seen for two participants (0.7%) in the overall 14-item scale and six participants (2%) in the overall 7-item scale (Table 3). There were no floor effects for any of the items of the scales (Table 3). The scores were skewed with skewness and kurtosis for each item ranging from (-7.2) to (-2.8), and 0.3 to 3.8, respectively.

**Table 3:** Internal consistency reliability of the Sinhala version of WEMWBS

Item	Floor effect N(%)	Ceiling effect N(%)	Item-total Correlation Coefficient	Cronbach’s alpha if item deleted (14-item scale)	Cronbach’s alpha if item deleted (7-item scale)
I’ve been feeling optimistic about the future^a^	14 (4.8)	49 (16.7)	0.50	0.90	0.83
I’ve been feeling useful^a^	5 (1.7)	71 (24.1)	0.66	0.89	0.80
I’ve been feeling relaxed^a^	19 (6.5)	62 (21.1)	0.59	0.90	0.82
I’ve been feeling interested in other people	7 (2.4)	75 (25.5)	0.38	0.90	
I’ve had energy to spare	13 (4.4)	60 (20.4)	0.62	0.89	
I’ve been dealing with problems well^a^	7(2.4)	82 (27.9)	0.70	0.89	0.80
I’ve been thinking clearly^a^	5 (1.7)	67 (22.8)	0.68	0.89	0.80
I’ve been feeling good about myself	10 (3.4)	109 (37.1)	0.64	0.89	
I’ve been feeling close to other people^a^	7 (2.4)	55 (18.7)	0.59	0.90	0.82
I’ve been feeling confident	6 (2.0)	120 (40.8)	0.69	0.89	
I’ve been able to make up mind about things^a^	6 (2.0)	59 (20.1)	0.66	0.89	0.81
I’ve been feeling loved	7 (2.4)	79 (26.9)	0.57	0.90	
I’ve been interested in new things	8 (2.7)	85 (28.9)	0.50	0.90	
I’ve been feeling cheerful	10 (3.4)	62 (21.1)	0.70	0.89	

^a^ Items in the 7-item scale

#### Construct validity

**Fig. 1:**
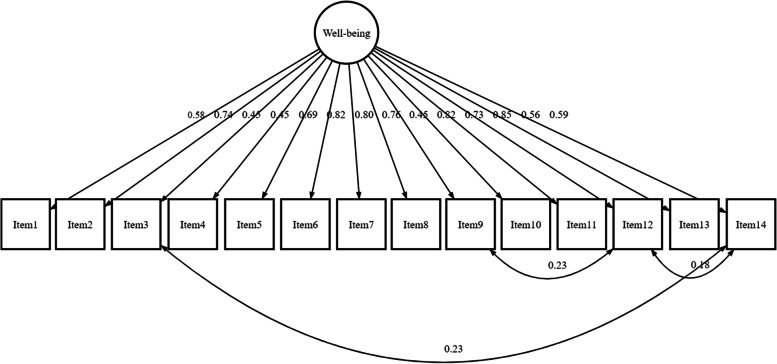
Path diagram of a single factor model of the 14-item version using confirmatory factor analysis

Using confirmatory factor analysis to test a one-factor (unidimensional) model, the chi-square fit index was significant (${X}_{77}^{2}$=326.63, *p* < 0.001) but RMSEA = 0.10; TLI = 0.93; and CFI = 0.94 indicated poor fit (Fig. 1). Factor loadings were > 0.4. Model fit was significantly improved after post-hoc modifications with fit indices of ${X}_{74}^{2}$=200.95, *p* < 0.001; RMSEA = 0.07 [95%CI 0.06–0.08]; TLI = 0.96; CFI = 0.97; and SRMR = 0.05 (Fig. 1).

**Fig. 2:**
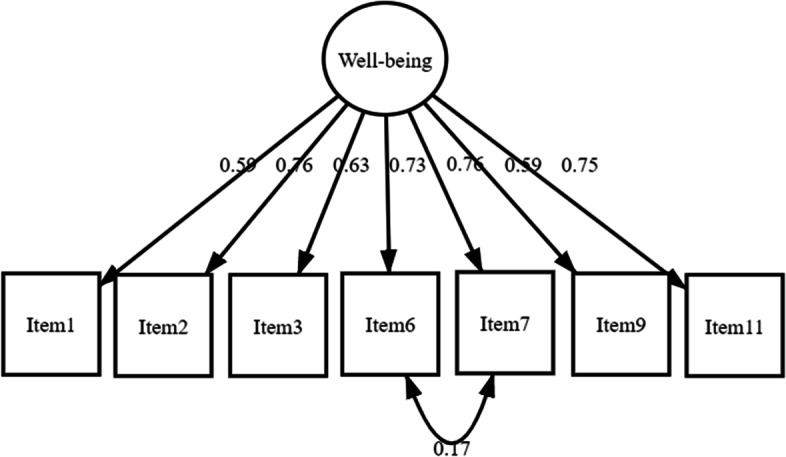
Path diagram of a single factor model of the 7-item version using confirmatory factor analysis

For the 7-item version, the fit indices were: Chi-square fit index ${X}_{14}^{2}$=59.40, *p* < 0.001; RMSEA = 0.11; TLI = 0.96; CFI = 0.97 and SRMR was 0.04. All factor loadings were > 0.5. After incorporating the post-hoc modifications, the final model had fit indices of $\mathrm{X}_{13}^{2}$=37.94, *p* < 0.001; RMSEA = 0.08 [95%CI 0.05–0.01], TLI = 0.97; CFI = 0.98; and SRMR = 0.03 (Fig. 2).

#### Internal consistency reliability

There was high internal consistency in both the 14-item (Cronbach’s alpha 0.90) and the 7-item scales (Cronbach’s alpha 0.83). Corrected item-total correlations ranged from 0.38–0.70 which were within the acceptable range [51]. The correlation between item 4 “I’ve been interested in other people” and the total score was at the lower end (Table 3).

#### Convergent validity

As hypothesized, WEMWBS scores were positively correlated with WHO-5 scores (Spearman *r* = 0.67, *p* < 0.001) and negatively correlated with PHQ-9 (Spearman *r* = -0.45, *p* < 0.001) and K10 (Spearman *r* = -0.54, *p* < 0.001) scores.

#### Known group validity

There were significant differences in the scores of both the 14-item and the 7-item scales between age groups, educational status and employment status (Table 4). WEMWBS scores were not associated with gender, ethnicity and religion. Scores of both versions of the scale were significantly different in persons likely to have depressive symptoms using a PHQ-9 cut-off of 10, and those likely to have psychological distress using a K10 cut-off value of 16 as compared to persons with better mental health status (Table 4).

**Table 4:** Association between WEMWBS total scores and selected variables

Variable	14-item scale			7-item scale		
	**Mean (SD)**	**Median (IQR)**	***P*-value**	**Mean (SD)**	**Median (IQR)**	***P*-value**
**Gender**						
Male (*n* = 88)	52.9 (9.2)	53.5(48–61)	0.67^a^	26.2 (4.8)	26 (24–30)	0.66^a^
Female (*n* = 202)	52.4 (9.4)	53 (47–60)		25.8 (4.9)	27 (23–29)	
**Age**						
15–24 (*n* = 79)	48.8 (10.2)	51(40–56)	< 0.001^b^	24.2 (5.2)	25.0 (20–28)	< 0.001^b^
25–44 (*n* = 159)	53.0 (8.8)	53 (48–59)		26.1 (4.5)	27.0 (24–29)	
45–74(*n* = 56)	56.5 (7.4)	57 (51–62)		27.8 (4.2)	28.0(26–31)	
**Religion**						
Buddhist (*n* = 208)	52.4 (9.3)	53(48–59)	0.54^a^	25.9(4.8)	27.0 (24–29)	0.83^a^
Other (*n* = 86)	52.9 (9.4)	54 (46–60)		25.9 (4.8)	27.0 (22–30)	
**Ethnicity**						
Sinhala (*n* = 283)	52.6 (9.3)	54 (48–60)	0.86^a^	25.9 (4.8)	27.0 (23–29)	0.70^a^
Tamil and Moor (*n* = 11)	51.6 (10.1)	53 (40–60)		25.1 (5.2)	26.0 (20–30)	
**Educational Level**						
School education only (*n* = 118)	54.2 (9.1)	55.5(50–61)	< 0.05^a^	26.6 (4.8)	27.5 (24–30)	< 0.05 ^a^
Higher education (*n* = 176)	51.4 (9.3)	53 (46–58)		25.5 (4.8)	26.0 (22.2–29)	
**Employment Status**						
Employed (*n* = 139)	54.0 (9.3)	55 (49–62)	< 0.001^b^	26.6 (4.8)	27 (24–30)	< 0.001^b^
Student (*n* = 92)	48.9 (9.6)	51 (41–55)		24.2 (4.9)	25.0 (21–27)	
Not-employed (*n* = 63)	54.6(7.3)	54.5(50–60)		26.8 (4.0)	27.0 (24–29)	
**PHQ scores**						
Score < 10 (*n* = 230)	54.3 (7.7)	54.5(50–60)	< 0.001^a^	26.8 (4.0)	27 (24–30)	< 0.001^a^
Score ≥ 10 (*n* = 64)	46.1 (11.4)	47.5(38–53)		22.6 (5.9)	22.5(19–26.7)	
**K10 scores**						
Score < 16 (*n* = 226)	55.1 (7.6)	55 (50–61)	< 0.001^a^	27.2 (3.9)	27.0 (25–30)	< 0.001^a^
Score ≥ 16 (*n* = 68)	44.1 (9.7)	43.5 (38–51.7)		21.7 (5.2)	21.5 (18.2–25)	

^a^ Mann Whitney U Test

^b^ Kruskal Wallis Test

#### Test–retest reliability

Test–retest reliability within 1–2 weeks was adequate (14-item scale - Spearman *r* = 0.72, *p* < 0.001; ICC 0.85, *p* < 0.001: 7-item scale - Spearman *r* = 0.72, *p* < 0.001; ICC 0.835, *p* < 0.001).

## Discussion

A culturally adapted Sinhala translation of the WEMWBS was developed and tested in a community sample in Sri Lanka. The Sinhala version of WEMWBS had acceptable psychometric properties indicating that it can be used as a programme monitoring tool to assess mental well-being at a population level and to evaluate large and small scale interventions. It is important for countries to be able to compare, set standards and targets for future Mental Health programmes [8, 52]. The validated Sinhala version of the WEMWBS, especially the short 7-item scale, is well suited for this purpose.

The strengths of the study are that we followed accepted methods for translation [53, 54] ensuring content validity, and obtained satisfactory results for reliability and construct validity. In our sample, some ceiling effects were found in individual items but not in the mean score of the scales. The item-total correlation of item 4: “I’ve been interested in other people” was comparatively low (0.39). Similar findings have been reported previously: in the Spanish validation, the correlation of item 4 was 0.44 [11]; it was 0.39 and 0.46 among Chinese students [28] and among health professionals in Pakistan [19], respectively. It appears that the concept of “interest in other people’ is perceived differently depending on the culture and norms of the study population.

Our sample of 294 was adequate both for CFA and for Structural Equation Modelling (SEM), where a sample size of 300 has been suggested to provide a good approximation of the chi square statistic and accurate standard errors when using the robust Diagonally Weighted Least Squares (DWLS) estimator for CFA models [55, 56]. While WEMWBS-Sinhala showed unidimensional characteristics with DWLS as reported in the original scales, further validations in other settings with larger populations are recommended to confirm these findings. As in this study, others have used post-hoc modifications in CFA to obtain acceptable fit indices [11, 24, 28, 29, 31]. We included three residual covariances with the 14-item scale and one for the 7-item short-version (WEMWBS) to obtain satisfactory fit indices.

The median mental-well-being scores of the Sinhala speaking Sri Lankan population were 53.5 and 27 for the 14-item and 7-item scales, respectively. This is much lower than the scores reported for general population samples in Spain [13] but higher than that for northern Ireland [57]. Our scores are comparable to the scores reported from Denmark and Austria [31, 58]. There was no significant difference in scores based on gender, ethnicity and religion. In our sample, the mean WEMWBS score increased with age. Older persons had better mental well-being [44]. Advancing age is related to an increase in the capacity to regulate emotions and the expression of more positive affections and lower level of negative affections [59]. The hedonic aspect of well-being improves with advancing age. The probable theory to explain these findings is the socio-emotional selectivity theory which suggests that as people age, they accumulate emotional wisdom that leads to selection of more emotionally satisfying events, friendships, and experiences. Contrary to our *a priori* hypothesis, based on Diener (1984), those who pursued higher education beyond grade 13 had lower well-being scores compared to those who had a school education only. In the UK, higher well-being scores were reported among those with higher education [9], similar to findings in Hong Kong [60]. One possible explanation in the Sri Lankan setting may be that with higher education, higher expectations are set which are more difficult to attain which may make people generally unhappy. However, there were no differences in PHQ-9 and K10 scores between those with and without higher education. Further research is indicated to tease out any mediating factors.

In our study, both gainfully employed and not-employed persons had higher well-being scores as compared to students, similar to findings of a study in Hong Kong [60]. Paid employment is argued to be important for individuals’ well-being because it provides an income and fulfills various psychological needs [61]. In Hong Kong, WEMWBS scores of those “not employed”, consisting mostly of homemakers, were not significantly different from those who were employed [60]. Homemakers can easily compensate for the loss or lack of financial benefits as they have a clear and important role of taking care of a household and children.

In our study, the student group had the lowest well-being scores as well as the highest depression and psychological distress scores. A study that explored mental health of university students across 26 Asian countries found high rates of moderate and severe depression among students [62]. The majority of our sample of students was following fairly strenuous medical and healthcare related professional courses. Distress rates of medical students in Sri Lanka are higher than those reported among students in other countries, which may be part of the explanation [63].

We chose to validate a scale developed in another cultural setting for many reasons. Firstly, the WEMWBS has been translated and culturally validated in many countries and settings [9, 11, 18, 21, 23–29]. Secondly, it contains only positive items which are easily understood by the participants. Third,the use of an already cross-culturally validated tool enables inter-cultural comparisons [64].

This study had a few limitations mainly due to the nature of the sample. Firstly, there was a high percentage of females (68.7%), and 31.3% were students. Secondly, the majority of the respondents was from one province (Western) with has predominantly urban and semi-urban populations and the findings cannot be generalised to the country. As responses to the questions of the scale are likely to be influenced by cognitive ability [51], the tool should be tested in more rural sub-populations. The online data collection done using snowballing may have introduced sampling bias. We used an “etic” approach to identify and adapt a well validated measure of well-being ( the WEMWBS) for a number of pragmatic reasons. This does not preclude future study to further test how the Sinhala speaking population conceptualises and expresses well-being. As highlighted in Henrich, Heine &Norenzayan’s (2010) seminal paper on WEIRD people, scales developed and tested in the Western cultures may not accurately assess the given construct in non-western cultures [65]. Even the items that are meant to capture what constituted as well-being in a western culture may not be seen important in a non-western culture. There is room for future studies to investigate how non-western cultures define well-being and to evaluate to which extent that their idea of well-being is captured by the WEMWBS.

## Conclusion

The culturally adapted 14- and 7-item versions of the Sinhala WEMWBS have acceptable psychometric properties. They can be used as a mental well-being assessment tool among the Sinhala speaking populations in Sri Lanka. From a public health perspective, the availability of a psychometrically robust tool allows monitoring population mental well-being and in monitoring and assessing the effectiveness of mental health promotion programmes in Sri Lanka.

## Supplementary Information

**Additional file 1: Supplementary Table 1.** Mental status of the participants (*N*=294).

## Data Availability

The datasets generated and/or analysed during the current study are available in the Figshare.com repository. https://figshare.com/articles/dataset/WEMWBS_Sinhala_Dataset/20439318

## Abbreviations

WEMWBS: Warwick Edinburgh Mental Well-being Scale
WHO-5: World Health Organization Well-being Index
PHQ-9: Patient Health Questionnaire
K10: Kessler Psychological Distress Scale
SEM: Structural equation modeling
DWLS: Diagonally weighted least squares
CFA: Confirmatory factor analysis
ICC: Intraclass correlation coefficient
RMSEA: Root mean square error of approximation
CFI: Comparative fit index
TLI: Tucker Lewis Index
SRMR: Standard root mean squared residual
IQR: Interquartile range
