# Supplementary material for: Measuring mental well-being in Sri Lanka: validation of the Warwick Edinburgh Mental Well-being Scale (WEMWBS) in a Sinhala speaking community
Source: BMC Psychiatry. 2022 Aug 24;22:569. doi: 10.1186/s12888-022-04211-8 (PMC9400250; doi:10.1186/s12888-022-04211-8)
Supplement: Supplementary file 1 — Additional file 1: Supplementary Table 1. Mental status of the participants (N=294). [file 12888_2022_4211_MOESM1_ESM.docx]

|  | **Mean Scores** | | | | | |
| --- | --- | --- | --- | --- | --- | --- |
|  | **WHO-5** | **p value** | **PHQ-9** | **p value** | **K10** | **p value** |
| **Gender** |  |  |  |  |  |  |
| Male | 13.4 (6.2) | 0.283^a^ | 5.9 (4.7) | 0.802^a^ | 9.5 (7.4) | 0.364^a^ |
| Female | 14.3 (5.8) |  | 6.1 (4.9) |  | 10.5 (7.8) |  |
| **Highest education** |  |  |  |  |  |  |
| School education only | 15.1 (5.8) | <0.01^a^ | 6.6 (5.6) | 0.332^a^ | 10.1 (8.3) | 0.579^a^ |
| Higher education | 13.3 (5.8) |  | 5.7 (4.3) |  | 10.2 (7.2) |  |
| **Occupation** |  |  |  |  |  |  |
| Working | 14.8 (5.7)^c^ | 0.001^b^ | 4.7 (3.5)^c^ | <0.001^b^ | 9.1 (7.2)^c^ | <0.05^b^ |
| Student | 12.0 (5.9)^d^ |  | 7.5 (5.1)^d^ |  | 11.7 (7.3)^d^ |  |
| Not working | 15.1 (5.7)^c^ |  | 6.9 (6.1)^d^ |  | 10.2 (8.8)^c,d^ |  |

Supplementary Table 1-Mental status of the participants (N=294)

a- Mann Whitney U Test, b- Kruskal Wallis Test

c,d - Means having a superscript with the same letter are similar
